# Autophagy and longevity: Evolutionary hints from hyper-longevous mammals

**DOI:** 10.3389/fendo.2022.1085522

**Published:** 2022-12-20

**Authors:** Andrea G. Locatelli, Simone Cenci

**Affiliations:** ^1^Age Related Diseases, San Raffaele Scientific Institute, Division of Genetics and Cell Biology, Milano, Italy; ^2^University Vita-Salute San Raffaele, Milano, Italy

**Keywords:** aging, ATG, autophagy, age-related diseases, bats, evolution, longevity, mitochondria

## Abstract

Autophagy is a fundamental multi-tasking adaptive cellular degradation and recycling strategy. Following its causal implication in age-related decline, autophagy is currently among the most broadly studied and challenged mechanisms within aging research. Thanks to these efforts, new cellular nodes interconnected with this phylogenetically ancestral pathway and unexpected roles of autophagy-associated genetic products are unveiled daily, yet the history of functional adaptations of autophagy along its evolutive trail is poorly understood and documented. Autophagy is traditionally studied in canonical and research-wise convenient model organisms such as yeast and mice. However, unconventional animal models endowed with extended longevity and exemption from age-related diseases offer a privileged perspective to inquire into the role of autophagy in the evolution of longevity. In this mini review we retrace the appearance and functions evolved by autophagy in eukaryotic cells and its protective contribution in the pathophysiology of aging.

*“Nothing in biology makes sense except in the light of evolution”*

Theodosius Dobzhansky, 1972

## Introduction

Autophagy encompasses chaperone-mediated autophagy, microautophagy, and macroautophagy, with the latter accounting for most autophagic activity, hence conventionally being referred to as autophagy. In this biological process, cells digest supramolecular structures through their isolation within specialized vesicles and the subsequent delivery of engulfed cargos to the lytic organelle, either the lysosome or the lytic vacuole, respectively in animal and plant or yeast cells (1, 2). Autophagy serves multiple fundamental functions, acting as a quality control mechanism affording renewal of dysfunctional compartments or unwanted structures, as well as a recycling hub supplying bioenergetic substrates and anabolic equivalents, either constitutively or in response to extrinsic and intrinsic stress. As a result, autophagy is a vital strategy conferring adaptive capacity to maintain cellular homeostasis (1, 3). Canonically, autophagic substrates, such as protein aggregates and damaged organelles, are degraded upon ubiquitin-like conjugation and recruitment of receptor/adapter proteins that afford adherence of the growing autophagic vesicle - called *phagophore* when still elongating, and *autophagosome* when complete - around the target (4). The prototypic and most thoroughly investigated receptor is SQSTM1/p62, hence the term SQSTM1-like receptors (SLR) to indicate the group of functionally related proteins hitherto identified (5, 6). Alternatively, organelles may be autophagocytosed through membrane-inbuilt receptors that harbour at least one phagophore-recognizing domain (ATG8-interacting motif, AIM, or LC3-interacting region, LIR) in their cytosolic portion. Once the autophagosome is sealed around the cargo, this is swiftly (within minutes) dispatched to the lytic compartment for degradation and its chemical constituents recycled by the cell (7).

Since the initial recognition and description of autophagy, a wealth of specific functions and interactions with other cellular processes have been unveiled (8), warning against the oversimplification of complex biological processes. At the cellular level, autophagy carries out multiple functions, such as the selective degradation of invading pathogens (*xenophagy*), protein aggregates (*aggrephagy*), and virtually all organelles, such as the endoplasmic reticulum (ER), mitochondria, peroxisomes, even up to the nucleus (*ER-phagy*, *mitophagy*, *pexophagy*, *nucleophagy*, *etc.*) for homeostatic maintenance (9). Autophagy-related genes (ATGs) are also involved in non-degradative functions (10). This concept is epitomized by their role in *unconventional secretion*, indicating the diverse trafficking strategies that route leaderless proteins, *i.e.*, lacking an ER-targeting amino-terminal leader peptide, to the extracellular space, collectively referred to as “ATG-dependent secretion”, or, less congruously (being generally independent on autophagic digestion), “secretory autophagy” (11).

At the organismal level, we now acknowledge a distinctive involvement of autophagy in opposing aging and the onset and progression of age-related diseases (4, 5, 12), yet the mechanistic architecture behind this function is far from being well defined. It is thus necessary to ascend to the origin of this process to understand the evolutive frame in which autophagy shaped itself. Key information may be achieved by exploiting the available molecular evidence from long-lived biological models. Attesting to the added value of this approach, the longest-lived mammals are revealing peculiar regulation of autophagy-related genetic products supporting enhanced autophagic activity (13, 14). Controversially, modern studies linking autophagy with aging still largely rely on experimental setups in short-lived mammals such as mice or phylogenetically distant organisms such as yeasts and flies. While these experimental models have obvious advantages that include amenability to genetic manipulation, the potentially ground-breaking information hidden within genomes of long-lived, taxonomically close to human species should not be neglected. The conceptual definition of autophagy dates back to 1963, when it was firstly presented by Christian de Duve in a symposium on lysosomal activity as the process of delivering large intracellular content to the degradative organelle (15). Focusing on this prime function, here we will climb up the evolutive path to inquire into the contribution of autophagy to the evolution of longevity and protection from onset and progression of age-related diseases in hyper-longevous mammalian models.

## The evolution of autophagy

A primitive cell-autonomous mechanism affording both metabolic homeostasis and natural immune defense against intracellular pathogens, autophagy is endowed with high intrinsic plasticity (16). In an evolutionary perspective, this feature is typically associated with ancestral traits, as witnessed by highly conserved mechanisms such as innate immunity and programmed cell death (17, 18). The presence of the same autophagic machinery within taxonomically divergent organisms such as animals and plants further attests to its positive adaptive selection and ancient origin (19). We would then expect to find this process active across phylogenetically distant taxa, such as prokaryotes and eukaryotes. Several studies have sought the presence of autophagy in different cellular models across the phylogenetic tree in order to clarify its evolutive path (20–22).

Typically, autophagy can be observed by detecting intracellular topical double-membraned cargo-engulfing vesicles *via* electron microscopy, by monitoring the release of amino acids from labelled proteins (thus exploiting autophagic catabolic activity) or by establishing the presence of key autophagic genes across different genomes (23, 24). Early studies performed on yeast models allowed to identify a first set of 27 ATGs, later expanded to over 40, and to characterize their respective functions (25). The presence of ATG orthologs was then investigated across the eukaryotic domain, confirming that the process was active in the last eukaryotic common ancestor (LECA), tracking back the origin of autophagy to more than 2 billion years ago (21, 26, 27). From yeasts to mammals, autophagy is present throughout, exception made for few unicellular parasites belonging to the excavata and chromalveolata supergroups (20). In these organisms, the ATG12 subpathway and its function were lost during evolution, arguably as a consequence of a distinct adaptation to the parasitic niche they inhabit, which overcame the need for alternative sources of nutrients. As for the prokaryotic domain, the autophagic process appeared to be completely absent (28). This was explained by the tight dependence of autophagy upon the presence of a relatively large degradation-devoted organelle such as the lysosome or the lytic vacuole, which are instead absent within the prokaryotic domain (29, 30). Compartmentalized protein degradation remains, however, a crucial function for cell homeostasis, which in bacteria is accounted for by proteases organized in multi-subunit structures resembling the more complex proteasome found in Eukarya (20, 31). The existence of a supplementary catabolic machinery such as autophagy in eukaryotes was indispensable to deal with the degradation of membrane-bound organelles that characterize the higher level of complexity and functional diversification of such cells (32). Moreover, autophagy provides conjugation, recognition and transport machineries that enable to mobilize large structures for longer distances (33), being eukaryotic cells ~1,000 times bigger in volume than prokaryotic counterparts (34). Finally, the higher level of complexity of Eukarya likely favoured the evolutionary onset of a more refined system for general homeostasis and an alternative source of energy substrates in the dire straits of nutrient starvation in a more energy-dependent machinery than the prokaryotic counterpart (35).

**Figure 1:**
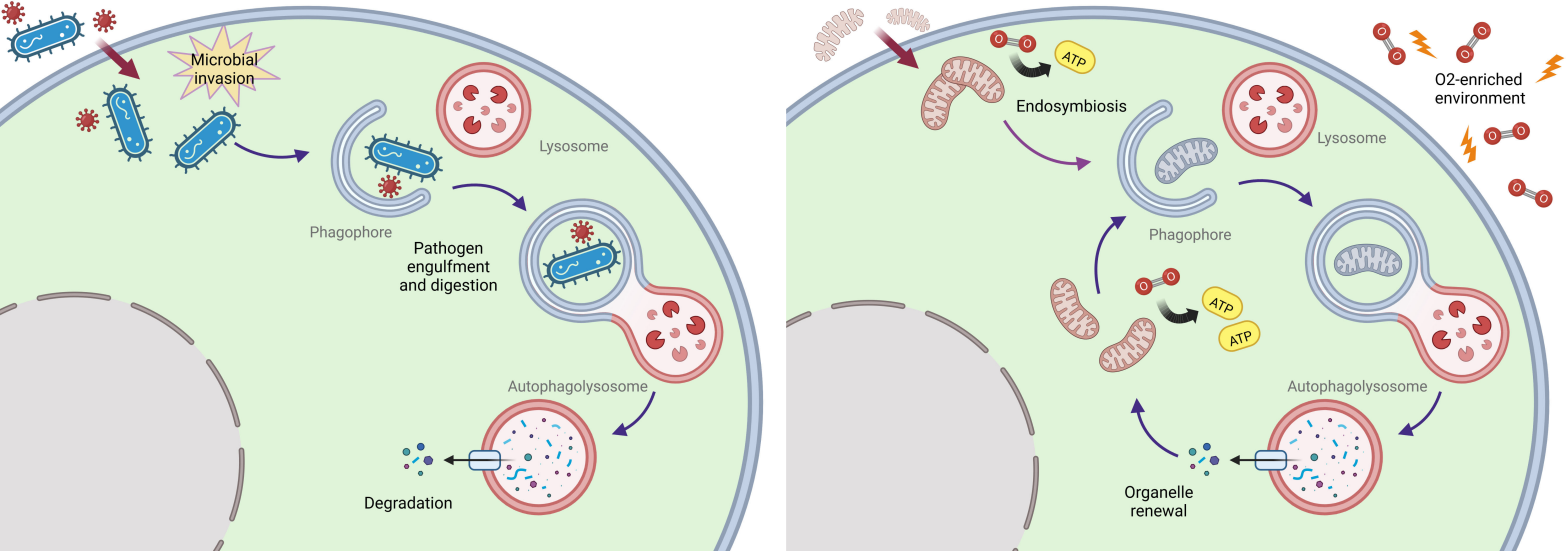
*Proposed endosymbiosis-driven origin of autophagy.* An ancestral immune defense strategy affording recognition and lysosomal clearance of intracellular pathogens (left) may have evolved into a fundamental selective organelle renewal pathway upon the integration of alpha-proteobacteria as mitochondria in eukaryotic cells under oxidative pressure (right).

The precise origin of autophagy was however still unclear, especially in view of its complexity and vast array of proteins involved. In a recent comparative genomic study, researchers were able to detect homologs of autophagy-related proteins in prokaryotes (especially in Cyanobacteria and Euryarchaeota) (22). Through this approach, they inferred a prokaryotic origin of the process and a following gain of function of a group of proteins originally devoted to distinct roles, as revealed by comparative genomics of multiple sequences encoding for autophagy-related genes. This was epitomized by ATG11, a selective autophagy adapter in yeast whose archaebacteria homologs are involved in DNA repair, chromosomal condensation, and partition. Notably, the same study also found that most ATG proteins are not expressed in the unicellular eukaryotic organisms, *Encephalitozoon cuniculi*, *Entamoeba histolytica*, and *Trypanosoma brucei*, previously found to lack most factors implicated in mitochondrial protein import (36). This finding reveals the symbiotic integration of Alphaproteobacteria in primordial eukaryotic cells as the driving force of autophagy evolution, which likely developed to afford quality control of the newly imported organelle (22, 37). Mitophagy might thus be assumed as the direct evolution of xenophagy, likely the most ancient form of innate immunity (16, 38, 39). In this view, bacteriophagy may have been co-opted to afford renewal of mitochondria, thereby ensuring survival under the critical selective pressure generated by the great oxygenation event (GOE) 2.5-2 billion years ago (16, 39, 40), (**Figure 1**).

Protein aggregates also represent ancestral noxious objects dealt with already in prokaryotes through disaggregating enzymes (41) and may have been among the first targets of this evolving selectiveness, as witnessed by the presence of an ancestral form of NBR1 in the LECA (42).

Coherently, while selective autophagy displays an uneven pattern of evolution among eukaryotes (43), mitophagy and aggrephagy are conserved across all the lineages of the domain (19, 44). Selective autophagy relies on the interplay between specific ubiquitin-like proteins and conjugation systems such as ATG8, ATG5 and ATG12 and selective autophagy receptors (SARs) harbouring ATG8-/ubiquitin-interacting motifs (AIM/UIM) (45, 46). Inhomogeneity in the presence of these receptors and relative homologs across the eukaryotic domain suggests that specialized forms of selective autophagy appeared later in evolution to deal with the renewal of organelles and afford cell type-specific homeostasis and survival (47).

Evidence of differential deployment of selective autophagy can be found in higher eukaryotes, as exemplified by the high rates of lysosomal digestion of different cargos across distinct cell types based on specific function and metabolic needs (9). Examples include mitophagy ensuring lifelong survival of post-mitotic stem cells (48) or ER-phagy affording proteosynthetic quality control in professional secretory cells (49, 50). In keeping with this view, higher number and specialization of SARs is observed in more complex eukaryotic organisms such as Metazoa and, at the same time, distinctive SARs have been identified in phylogenetically distant organisms, in line with the taxon-specific inferred selective pressures (*e.g.*, autophagic receptors for chloroplasts in plants) (43, 51, 52). Furthermore, an expanded SARs repertoire may also confer functional redundancy upon higher eukaryotes (53).

Protein architecture studies revealed that the paradigmatic yeast SAR, ATG19, harbours multiple AIMs, a sensible feature to discriminate supramolecular targets from single proteins destined to the proteasome for degradation (54). In contrast, metazoan receptors express single AIMs/UIMs and depend on a polymerization domain to crosslink the autophagic vesicle with the cargo (55). The evolution of a different target recognition strategy is associated with the presence of domains responsible for additional cytoprotective non-autophagic functions that may turn precious when adapters accumulate heralding insufficient or overwhelmed autophagic activity. Exemplary is the case of the KEAP1-interacting region harboured by p62, which is capable of stabilizing Nrf2 and activating the downstream antioxidant transcriptional response when p62 accumulates (56). Oxidative stress is a chief source of proteotoxicity; the resulting saturation of autophagy may then trigger a tailored antioxidant response.

Overall, autophagy appears to be a well conserved mechanism among eukaryotes, providing crucial abilities in terms of cell-specific organellar homeostasis maintenance, possibly co-opted from an ancestral mechanism of cell defense.

## Autophagy in long-lived mammalian taxa

The decline of autophagic ability is one of the most acknowledged molecular hallmarks of cellular aging (57). As eukaryotic organisms age, they suffer from a progressive, maladaptive decrease in the ability to activate autophagy and benefit from its degradative/renewal properties, leading the cells to accumulate damaged organelles and cytotoxic macromolecules overall (4, 12, 58). Autophagy appears to be intimately connected with the modulation of longevity, as proved by several studies which demonstrated an effect on cellular and organismal lifespan when autophagy was harnessed either genetically or pharmacologically (59–61). Loss of autophagy is also linked with the worsening of many age-related diseases such as neurodegeneration and cancer (62, 63). The exact mechanisms behind this connection are yet unclear, given the vastity of genes involved in the process and the different function afforded by autophagy including proteostasis, nutrient regulation and immunity (4).

Evolution provides us with evidence of selective adaptations in the autophagic process across long-lived organisms, including phylogenetically close-to-humans taxa belonging to the mammalian clade (59). This confirms the existence of an either direct or indirect link between autophagy and lifespan modulation but concurrently may represent a unique opportunity to shed light on the key molecular elements involved through comparative studies. A connection between autophagic activity and organismal lifespan was first identified in a pioneering study on insulin/IGF-1 signalling, where autophagy-inducing mutations in *daf-2* were associated with lifespan extension in *C. elegans* (64) and later confirmed in organisms such as drosophila, mice, and humans (14). Another important discovery linking autophagy with longevity emerged from studies on mTOR signalling and dietary restriction, an established universal life-extending intervention. Starvation-induced autophagy was proven to be causal to lifespan extension in several animal models from yeasts to great apes (65, 66). Moreover, direct inhibition with rapamycin of the mTOR kinase, a master nutrient-sensing regulator of autophagy, systematically increased the median and maximum lifespan of mice (60). The intimate correlation between autophagy and lifespan modulation was further confirmed in works on germline removal and lipid turnover (67), reactive oxygen species (68) and mitochondrial respiration (69). Additional evidence came from either direct or indirect pharmacological manipulation of autophagy *via* different drugs such as spermidine, resveratrol, tomatidine, urolithin-A, metformin, and dorsomorphin and from genetic manipulation of autophagy regulators such as TFEB, microRNAs (miR-34), sirtuins, and forkhead transcription factors (FOXO) (14).

**Figure 2:**
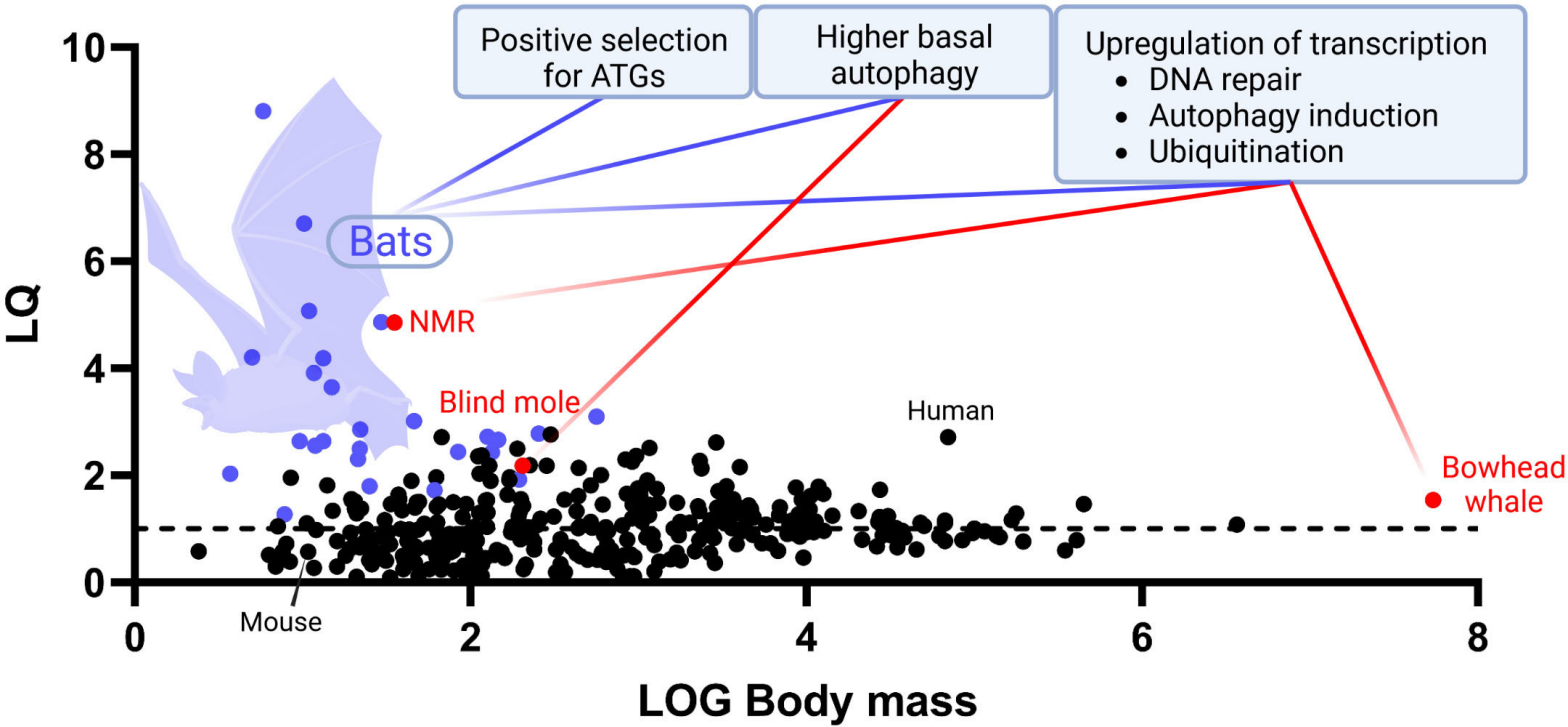
*Evolutive autophagy-associated adaptations underlying mammalian longevity.* An up-to-date graph representing the distribution of longevity quotients (LQ, Y axis) calculated for 352 species of mammals of different body mass (log, X axis). Boxes display documented evolutive adaptations of autophagy-related key molecular features that may contribute to explain taxon-specific enhanced longevity in bats (purple dots), NMR, blind mole, and bowhead whale (red dots). Maximum lifespan data were collected from AnAge longevity database, https://genomics.senescence.info/species/index.html. The graph is an updated version of the same firstly presented in Austad 2010 (71).

Once established, the correlation between autophagy and aging encouraged an upsourging number of scientific works over the last decade in both conventional and unconventional mammalian models hallmarked with different longevity. Transcriptomic studies of the longest-lived mammal, the bowhead whale (*Balaena mysticetus*), revealed overexpression of genes for DNA repair, autophagy induction and ubiquitination (70). To better inquire into the evolution of longevity in mammals, further studies were aimed towards the identification of unique adaptations in molecular markers of aging in taxa characterised by high *longevity quotients* (**Figure 2**). Indeed, among mammals, a direct correlation exists between maximum lifespan and size, with bigger organisms living more than small ones. The reasons behind this correlation are multiple, tightly linked with the evolution of reproductive patterns and sexual maturation. Nevertheless, some mammals lie outside this statistic, being able to live longer than expected, thus owning a higher-than-average longevity quotient (71). One of the most studied mammalian species characterised by a high longevity quotient is the naked mole rat (NMR, *Heterocephalus glaber*). This rodent is capable of living substantially more than expected more for a mammal of comparable body size, holding one of the highest quotients of its clade (72). Interestingly, studies of the NMR showed higher basal autophagic activity (measured as expression of LC3II and beclin-1 autophagic marker proteins) when compared with C57Bl/6 mice (73). Furthermore, NMR’s transcriptome analyses recapitulated features found in the bowhead whale, with overexpression of genes for DNA repair and autophagy, which proved down-regulated in mammals with low longevity quotients, such as mice and cattle (74). A study on the speciation of another noncanonical rodent model characterized by a high longevity quotient, the blind mole (*Spalax galili*), revealed a strong dependence on proteostatic machineries such as autophagy and the proteasome in determining niche adaptation, since these animals need to deal with a high metabolic stress deriving from the limited nutrient sources of soil dwelling (75). In this case, upregulation of autophagy appeared to have evolved as a consequence of a specific environmental pressure (*i.e.* scarcity of nutrients and the resulting need to deal with protein aggregates deriving from dwelling in nutrition-compromised chalk-rich soils). This may ultimately have set the conditions for a pro-longevity gain of function, arguably as an indirect consequence of the cytoprotective properties of enhanced autophagic activity. Another example of this phenomenon may be the case of the unique evolution witnessed in bats, the order of mammals with the highest longevity quotient among all (76). Bats (chiroptera) represent a one-of-a-kind study model for inquiring into the evolution of longevity in mammals, as they constitutively live longer than expected at a higher taxonomical level than other hyper-longevous species. This implies the existence of an evolutionarily conserved pro-longevity molecular feature embedded within their genome. During the last years, several studies have been aimed to decipher the exceptional resistance of bats against aging and age-related diseases with many of these reporting an upregulation of autophagic activity across different tissues when compared with mice and other mammals (77). In a study on primary fibroblasts, both young and aged bats were found to have a constitutively higher level of autophagic flux than murine counterparts. Further analyses on the blood transcriptome showed upregulation of autophagy-associated genes and transcript enrichment for terms associated with macroautophagy and positive regulation of autophagy. Finally, phylogenomic analyses detected evidences of positive selection acting on autophagy-associated genes (including ATG9B, LARP1, mTOR, MFN2, NPC1, STOM and VPS4A) in bats, thus indicating the existence of differential evolutive forces acting on autophagy in this taxon versus the rest of the mammalian clade (13). Recent publication of complete reference-quality genomes of 6 species of bats, achieved further genetic insights, highlighting once more the pivotal role of autophagy in the evolution of longevity within these mammals (78).

Autophagy in bats arguably evolved to face the massive production of cytotoxic metabolic by-products deriving from the extremely energetically demanding activity of powered flight, for which bats are the only performers among mammals (79). Likewise for what observed in blind moles then, following positive selection of the autophagic process for its homeostatic maintenance abilities, bats may have benefit of a gain of function in lifespan extension thanks to the pro-longevity properties of such mechanism. Evidences of the link between coevolution of pro-homeostatic activity and longevity in flight are provided also by studies on flying and non-flying birds, with flying ones living longer and displaying higher levels of autophagy overall (80, 81). However, also thanks to their taxonomical rank, bats are currently becoming one of the most studied alternative biological models for better understanding the evolutionary intake of autophagy on lifespan extension in mammals (**Figure 2**).

Finally, being aging now acknowledged as the driving cause of all age-related disorders, worth of interest are the evolutionary implications deriving from evidence of resistance from these diseases in the longest-lived mammalian models. Further attesting to autophagy as an anti-aging biological asset, recent studies found high autophagic activity to inversely correlate with the incidence and severity of pathologies associated with aging, such as neurodegeneration, frailty, and cancer (1, 4, 12, 82). Bats are once again a unique study model as they show high cognitive performances (*e.g.*, echolocation) throughout their extended lifespan (83), do not display phenotypic aging (young and old bats are macroscopically indistinguishable) (84), and show lower occurrence of cancer when compared with other mammals (85). Several studies are currently attempting to gauge the exact contribution of autophagy to disease protection and to address and dissect the underlying cellular and molecular mechanisms in these models (86, 87). Given our taxonomic proximity to bats and the other hyper-longevous mammals, these studies are likely to complement investigations employing canonical animal models, towards a deeper understanding of the role of autophagy in opposing aging and age-related diseases in humans.

## Conclusions and perspectives

Autophagy is an evolutionarily ancestral process that developed within eukaryotic cells likely as a consequence of adaptations driven by the mitochondrial endosymbiotic event building on molecular cues of intracellular innate immunity. Following its early appearance, roughly 2 billion years ago, autophagy underwent several adaptive gains of function, at first in terms of substrate selectiveness within specific intracellular environmental niches (*e.g.*, chloroplasts in plant cells, or secretory granules in professional secretory cell types) and then as a homeostatic strategy ultimately promoting organismal lifespan extension. The tight connection existing between autophagy and aging is testified by several model organisms, where prolonged lifespan typically correlates with enhanced activity of autophagy and relative regulatory pathways. Evolutionary evidence of this relationships can be found in many long-lived vertebrates and, of translational relevance, in mammals such as whales (featuring enhanced lifespans) and mole rats and bats (featuring outstanding longevity quotients).

A growing number of scientific advances across different disciplines benefit from an evolutionary unbiased perspective that outlives our understanding of life sciences. Pursuing the molecular development of autophagy since its early form is a valuable strategy to infer new hierarchies in the mechanisms involved and to devise effective therapeutic applications. Comparative-correlative studies on unconventional mammalian models of extraordinary longevity are already hinting at novel targets of strategic relevance to harness autophagy and treat age-related diseases. Investing along this new research attitude is necessary to overcome our limitations in the understanding of complex biological processes such as autophagy and its implications in the evolution of longevity.

## Author contributions

All authors contributed to the article and approved the submitted version.
